# Predicting substituent effects on activation energy changes by static catalytic fields

**DOI:** 10.1007/s00894-017-3559-6

**Published:** 2017-12-22

**Authors:** Martyna Chojnacka, Mikolaj Feliks, Wiktor Beker, W. Andrzej Sokalski

**Affiliations:** 1 Advanced Materials Engineering and Modelling Group, Faculty of Chemistry, Wrocław University of Science and Technology, Wyb. Wyspiańskiego 27, 50-370 Wrocław, Poland; 2 Department of Chemistry, University of Southern California, Los Angeles, CA USA

**Keywords:** Substrate assisted catalysis, Catalytic fields, Fluorine substitution, Ab initio

## Abstract

Catalytic fields illustrate topology of the optimal charge distribution of a molecular environment reducing the activation energy for any process involving barrier crossing, like chemical reaction, bond rotation etc. Until now, this technique has been successfully applied to predict catalytic effects resulting from intermolecular interactions with individual water molecules constituting the first hydration shell, aminoacid mutations in enzymes or Si→Al substitutions in zeolites. In this contribution, hydrogen to fluorine (H→F) substitution effects for two model reactions have been examined indicating qualitative applicability of the catalytic field concept in the case of systems involving intramolecular interactions.

**Electronic supplementary material:**

The online version of this article (10.1007/s00894-017-3559-6) contains supplementary material, which is available to authorized users.

**Graphical abstract:**
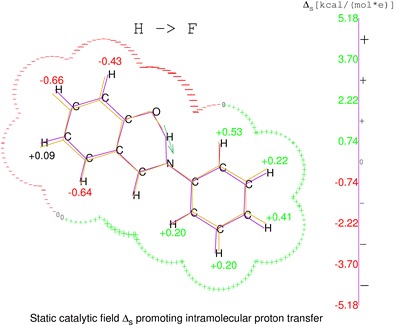
Hydrogen to fluorine (H→F) substitution effects on activation energy in [kcal/mol]

## Introduction

Recent experimental studies confirm the catalytic role of external electric fields [1–4]. This observation is in agreement with previous theoretical studies indicating the electrostatic nature of enzymatic or zeolite catalysis [5–11]. The catalytic activity measured as the lowering of the activation barrier *∆*, is dominated in many macromolecular systems (C), for example enzymes [8–10] or zeolites [11], by their electrostatic interactions with corresponding reactants, i.e., transition states E(C.TS) and substrates E(C.S). In the present contribution, we have used catalytic fields [7] derived as the difference of the MEP of corresponding transition states and substrates in an attempt to estimate catalytic effects resulting from H->F substitutions for two model reactions. This is in line with the study of Prof. Peter Politzer and coworkers [12], where the relationship of molecular electrostatic potentials (MEP) with experimentally based reactivity indices was postulated earlier.

## Methods

The catalytic activity can be estimated by using a variety of computational methods, for example by the differential transition state stabilization approach [7–11] (DTSS) representing activation barrier lowering (*∆*) in terms precisely defined within the theory of intermolecular interactions. In most cases, when DTSS is dominated by electrostatic interactions, *∆* could be approximated by a sum of products of molecular environment point charges q_i_^C^ and the difference of molecular electrostatic potentials of the corresponding transition state and substrate (V_i_^TS^ – V_i_^S^) [7–9]. The sum runs over all atomic point charges representing the molecular environment of the reaction being studied.

1 $$ \varDelta =\mathrm{E}\left(\mathrm{C}.\mathrm{TS}\right)\hbox{--} \mathrm{E}\left(\mathrm{C}.\mathrm{S}\right)\approx {\mathrm{E}}_{\mathrm{E}\mathrm{L}}\left(\mathrm{C}.\mathrm{TS}\right)\hbox{--} {\mathrm{E}}_{\mathrm{E}\mathrm{L}}\left(\mathrm{C}.\mathrm{S}\right)\approx \sum {{\mathrm{q}}_{\mathrm{i}}}^{\mathrm{C}}\left({{\mathrm{V}}_{\mathrm{i}}}^{\mathrm{TS}}\hbox{--} {{\mathrm{V}}_{\mathrm{i}}}^{\mathrm{S}}\right) $$

Due to the perfect additivity of the electrostatic term, one may represent the entire molecular environment by assembly of atomic point charges. In such a case, the activation barrier lowering by a single unit (positive or negative) charge amounting to

2 $$ {\varDelta}_{\mathrm{s}}=\hbox{--} \left({{\mathrm{V}}_{\mathrm{i}}}^{\mathrm{TS}}\hbox{--} {{\mathrm{V}}_{\mathrm{i}}}^{\mathrm{S}}\right) $$

is called a static catalytic field *∆*_s_. Assembly of *∆*_s_ represents a distribution of a hypothetical molecular environment expressing optimal catalytic activity for the reaction of interest [9–11]. Such a catalytic field constitutes the solution of the inverse problem of catalysis. One obtains in the single computational step, requiring only the knowledge of superimposed transition state and substrate electrostatic potential maps, the complete characteristics of the molecular environment charge distribution exerting optimal catalytic activity. Molecular electrostatic potentials V_i_^TS^ and V_i_^S^ could be efficiently estimated using cumulative atomic multipole moments obtained directly from wavefunctions of reactants [13].

In addition to external (intermolecular) fields, the catalytic activity can also be modified by intramolecular effects (for example substitutions or deletions of functional groups), which is reminiscent to the well-known phenomenon of substrate assisted catalysis [14]. In this type of catalysis, substrate substituents are introduced to modulate the catalytic activity instead of an external molecular environment.

**Scheme 1:**
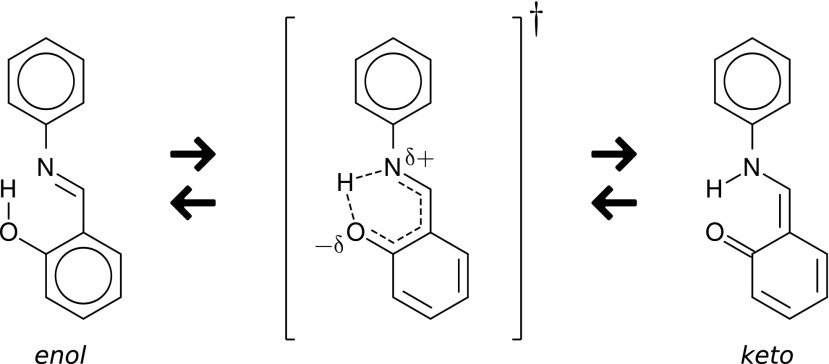
Proton transfer in salicydene aniline

**Scheme 2:**
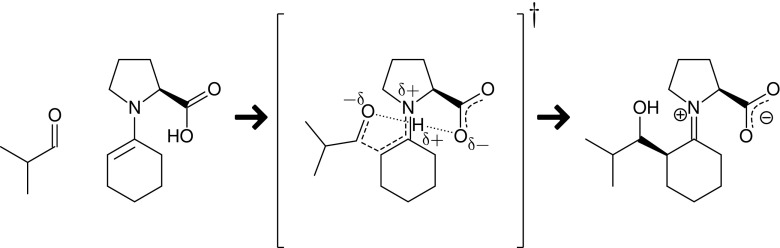
Addition of cyclohexanone to isobutyraldehyde catalyzed by L-proline

In the following report, we have examined two model systems, where catalytic fields, ∆_s_, have been confronted with changes of the activation energy resulting from the substitution of hydrogen by fluorine. In the first model system, a proton transfer in salicydene aniline [2] has been considered (Scheme 1):

The second reaction considered here is an asymmetric aldol reaction of cyclohexanone and isobutyraldehyde, catalyzed by L-proline [15] (Scheme 2).

As a starting point for our calculations, we used geometries of the transition states from the previous study by Bahmanyar and coworkers [15]. Frequency analyses were performed on the optimized geometries to verify the nature of reactant and transition states. Calculations of minimum energy paths (MEP) were done to generate substrate states from the transition states. The MEP calculations were performed using the intrinsic reaction coordinate algorithm (IRC). For all calculations, the B3LYP density functional theory method was used together with the 6-31G(d) basis set. For each substitution site (a hydrogen atom), a calculation of the electrostatic potential was done at a distance of 1.5 A from the site. In what follows, we focus on reaction “6a” from the previous study [15].

The first model represents a simple single proton transfer reaction in a planar aromatic molecule and the second one involves a much more complex non-planar organocatalytic molecular system.

## Results and discussion

In the first model case, we studied tautomeric enol-keto or keto-enol rearrangements within salicydene aniline, a model system representing a larger class of molecular switches [2]. These switches have become a “hot-topic” thanks to their potential application in molecular electronics, data storage and processing devices. In this context, it is vital to understand the role of functional groups introduced to salicydene aniline, as these may modify the energetics of the intramolecular keto-enol tautomerizations and isomerizations.

**Fig. 1:**
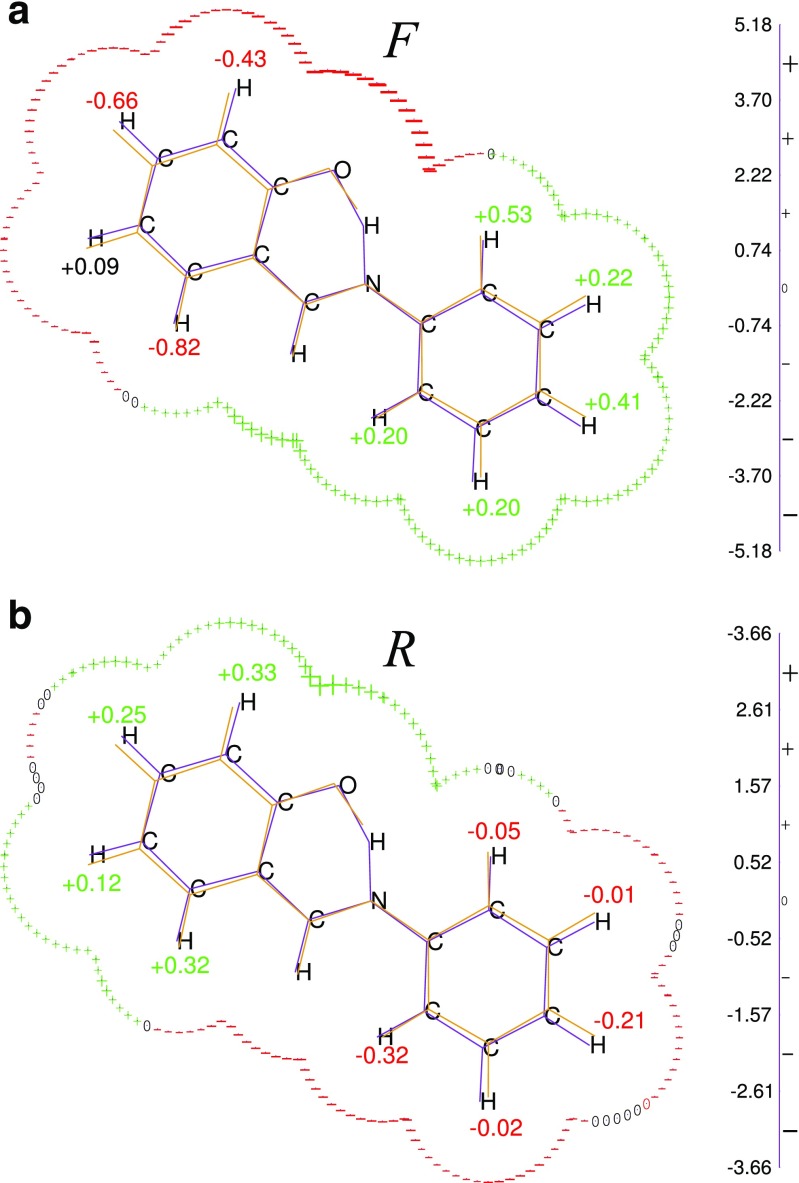
Catalytic field values ∆_s_, (in [kcal mol^-1^*e]) for the proton transfer in tautomeric rearrangements of salicydene aniline: enol→keto (“F”, forward) and keto→enol (“R”, reverse). The size of the “+” or “–” sign indicates the magnitude of the static catalytic field *∆*_s_, resulting from a single unit point charge located at the solvent accessible surface. Numerical values in [kcal mol^-1^] indicate lowering of the activation barrier Δ resulting from H->F substitution. Numerical values are given in Table S1 (Supplementary materials)

**Fig. 2:**
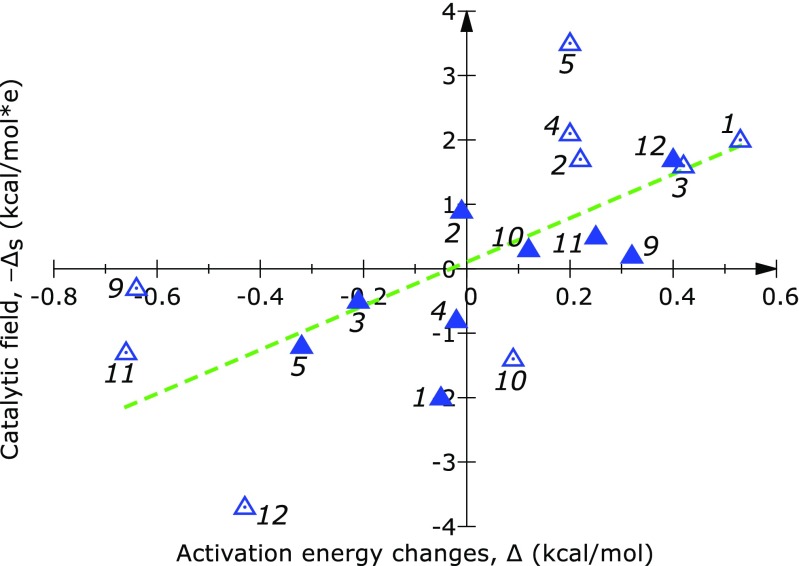
Relationship between the activation barrier lowering, Δ, of each fluorine substitutent and the catalytic field, Δ_s_, calculated at a position adjacent to each substitution site for tautomeric rearrangements of salicydene aniline. Empty and filled triangular marks indicate reactions in the forward and reverse direction, respectively. Numerical values are given in Table S1 (Supplementary materials)

We applied catalytic fields here to examine how the activation barrier for a proton transfer reaction could be modified by the intramolecular charge redistribution resulting from the substitution of various hydrogens by fluorine. The most pronounced changes of the activation barrier resulting from 16 H→F substitutions are in a qualitative agreement with predictions of the catalytic field, *∆*_s_ (Figs. 1 and 2). In two cases only (H_10_->F_10_ “F” and H_2_->F_2_ “R”) where a sign reversal is observed, *∆*_s_ values do not exceed 0.1 kcal mol^-1^*e. Due to the aromatic character of salicydene aniline and possible mesomeric effects, more quantitative agreement could not be possible. Overall, our calculations agree reasonably well with the alternative study of 2-hydroxy Shiff bases [16].

In the second case, we have considered the minimum energy reaction path for the aldol reaction of cyclohexanone with isobutyraldehyde catalyzed by L-proline [15]. The application of small organic molecules as catalysts in organic synthesis has seen a dramatic increase in recent years, as these catalysts are readily available, low-cost, and do not rely on metal cofactors, whose toxicity is often problematic in the production of pharmaceuticals [17]. Hence, there is a need for better understanding how these organocatalysts operate at the molecular level, which is where computational chemistry can provide some answers. Of particular interest is the knowledge of factors that control the stereoselectivity of asymmetric organocatalytic reactions, for example reactions catalyzed by proline.

**Fig. 3:**
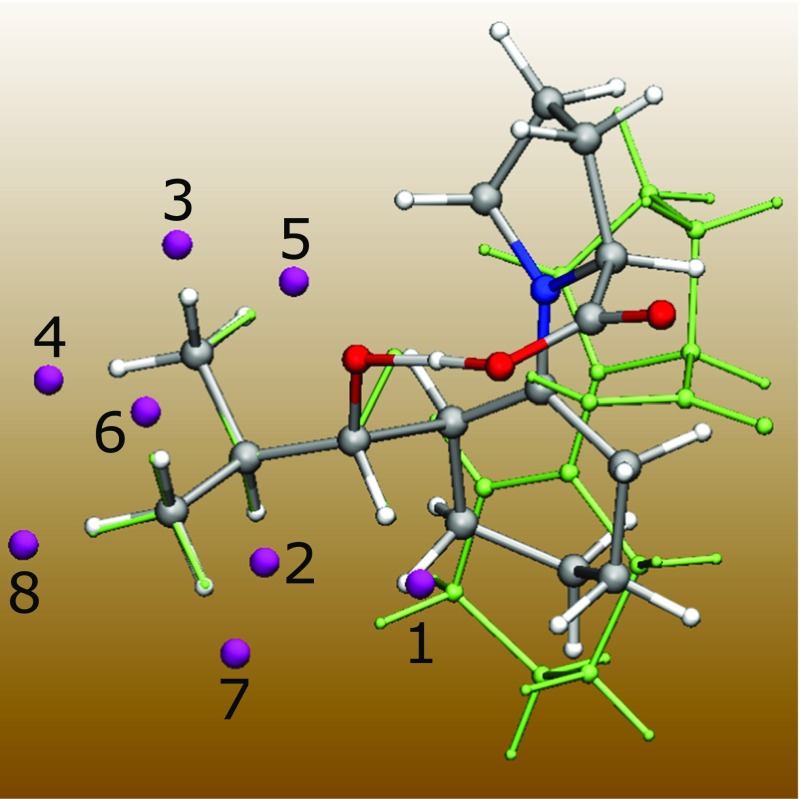
Superimposition of the optimized geometries of the transition (TS) and reactant state (RS) for the reaction of cyclohexanone with isobutyraldehyde catalyzed by L-proline. The TS and RS geometries are shown in multiple colors and green, respectively. Purple spheres indicate positions in space where a differential electrostatic potential is calculated. Each position is located at a distance of 1.5 Å from the corresponding substitution site (a hydrogen atom). The geometries were aligned so as to minimize the RMSD of the isobutyraldehyde atoms. The alignment was done in VMD [16]

**Fig. 4:**
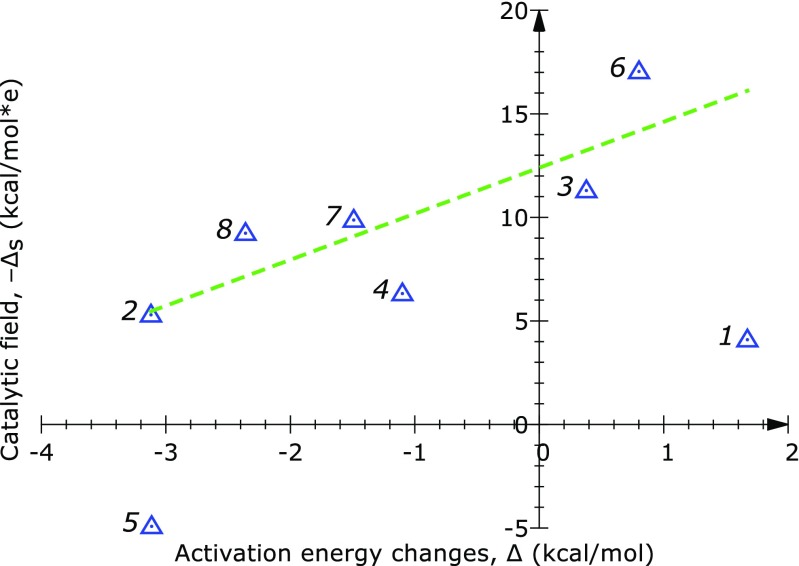
Relationship between the activation barrier lowering, Δ, of each fluorine substitutent and the catalytic field, −Δ_s_, calculated at a position adjacent to each substitution site (see Fig. 3 for the locations of these sites). Numerical values are given in Table S2 (Supplementary materials)

The effect of different H→F substitutions (Fig. 3) has been examined and compared with predictions of the catalytic field. As seen on Fig. 4 and Table S2, for the substituents at positions “2” to “4” and “6” to “8”, the dependency of the catalytic field on the reaction barrier is close to linear. Namely, the calculated standard deviation for these positions is as low as 2.2 kcal mol^-1^. This observation is promising, considering the fact that our approach is based on simple point potentials. However, the values of the catalytic field calculated at positions “1” and “5” do not seem to follow the linear trend. We note that the fluorine substitutents at these positions are located in the direct vicinity of the reacting region. For example, the hydrogen atom corresponding to position “1” is part of the reacting carbonyl group of the attacking isobutyraldehyde. Thus, substituents “1” and “5” may have the most pronounced effect on the energetics of the reaction. Moreover, at positions neighboring the reacting region effects other than electrostatic may be more dominating. If the electrostatic effects at these positions still play the major role, our description based on point potentials may in such cases be overly simplistic. Nevertheless, for substitutent positions away from the reacting parts of the system, we obtain a roughly linear relationship of the catalytic field and the calculated reaction barrier.

In both model cases, H→F substitution effects can be qualitatively predicted based on the values of the catalytic field calculated for distances of 1.5 Å from each substitution site. Some observed deviations from the linear relationship may be due to possible mesomeric effects, especially for the first model involving an aromatic system.

Recent experimental [18] and theoretical [19] studies on the effect of fluorine substitutions in O-GlcNAcase substrates provide additional evidence on the substrate assisted catalytic mechanism. The relationship between molecular electrostatic potentials and reaction rates was postulated earlier by Prof. Peter Politzer and coworkers [12, 20].

## Conclusions

In conclusion, static catalytic fields can be employed to predict with reasonable accuracy and at a low computational cost substitution effects on activation barriers. The use of catalytic fields is beneficial, since they allow in one computational step rapid evaluation of substituent effects. Usually, such effects are calculated by modeling of the reaction path of each substituted reactant, something that is a tedious and computationally expensive task. In our approach, however, it is sufficient to know only the geometries of the regular, non-substituted reactant and transition state. Once these geometries have been optimized, an “all-at-once” calculation of the electrostatic potential is performed at positions suitable for substitution. For each position, a difference of the electrostatic potential between the reactant and transition state can already provide a reasonable approximation of the corresponding substituent effect.

Since the calculation of catalytic fields is computationally inexpensive and can be for the most part automated, they may find application in areas, such as computer-aided drug design, molecular docking or virtual screening, where there is often a need for the evaluation of substituent effects in hundreds of molecules during a single study.

## Electronic supplementary material

ESM 1 (PDF 85 kb)
