# Supplementary material for: Predicting substituent effects on activation energy changes by static catalytic fields
Source: J Mol Model. 2017 Dec 22;24(1):28. doi: 10.1007/s00894-017-3559-6 (PMC5741779; doi:10.1007/s00894-017-3559-6)
Supplement: Supplementary file 1 — (PDF 85 kb) [file 894_2017_3559_MOESM1_ESM.pdf]

Predicting substituent effects on activation energy changes by static catalytic fields.

Supplementary Materials

Martyna Chojnacka,[1] Mikolaj Feliks,[2] Wiktor Beker,[1] W. Andrzej Sokalski[1,†]

[1)] Advanced Materials Engineering and Modelling Group, Faculty of Chemistry, Wroclaw University of Science and Technology, Wyb. Wyspianskiego 27, 50-370 Wroclaw, Poland

[2)] Department of Chemistry, University of Southern California, Los Angeles, CA, USA

[†)] correspondence author; e-mail: sokalski@pwr.wroc.pl

September 12, 2017

Table S1: Calculated activation energy barrier changes, Δ, and corresponding catalytic field $-\Delta_S$ values for the series of studied fluorine derivatives of salicydene aniline. The activation barrier for the unsubstituted substrate ("wildtype") was calculated to be 5.18 $^{\mathrm{kcal}}/_{\mathrm{mol}}$ for forward "F" and 1.02 $^{\mathrm{kcal}}/_{\mathrm{mol}}$ for reverse "R" proton transfer reaction, respectively.

Reaction	enol→keto (Forward)		keto→enol (Reverse)	
Substituent	Δ $[^{\mathrm{kcal}}/_{\mathrm{mol}}]$	$-\Delta_S$ $[^{\mathrm{kcal}}/_{\mathrm{mol}\cdot e}]$	Δ $[^{\mathrm{kcal}}/_{\mathrm{mol}}]$	$-\Delta_S$ $[^{\mathrm{kcal}}/_{\mathrm{mol}\cdot e}]$
1	0.5	2.0	−0.1	−2.0
2	0.2	1.7	0.0	0.9
3	0.4	1.6	−0.2	−0.5
4	0.2	2.1	0.0	−0.8
5	0.2	3.5	−0.3	−1.2
9	−0.6	−0.3	0.3	0.2
10	0.1	−1.4	0.1	0.3
11	−0.7	−1.3	0.3	0.5
12	−0.4	−3.7	0.4	1.7

Table S2: Activation barrier changes, Δ, and values of the catalytic field, $-\Delta_S$, for the series of studied fluorine derivatives of isobutyraldehyde. The activation barrier for the unsubstituted substrate ("wildtype") was calculated to be 14.0 $^{\text{kcal}}/_{\text{mol}}$.

Substituent	Δ $[^{\text{kcal}}/_{\text{mol}}]$	$-\Delta_S$ $[^{\text{kcal}}/_{\text{mol} \cdot e}]$
2	−3.1	−5.3
5	−3.1	4.9
8	−2.4	−9.2
7	−1.5	−9.9
4	−1.1	−6.3
3	0.4	−11.3
6	0.8	−17.0
1	1.7	−4.1
